# Re-construction layer effect of LiNi_0.8_Co_0.15_Mn_0.05_O_2_ with solvent evaporation process

**DOI:** 10.1038/srep44557

**Published:** 2017-03-20

**Authors:** Kwangjin Park, Jun-Ho Park, Suk-Gi Hong, Byungjin Choi, Sung Heo, Seung-Woo Seo, Kyoungmin Min, Jin-Hwan Park

**Affiliations:** 1 Energy Lab, Samsung Advanced Institute of Technology (SAIT), Electronic Materials Research Complex, 130 Samsung-ro, Gyeonggi-do, 16678, Republic of Korea; 2 Platform Technology Lab, Samsung Advanced Institute of Technology (SAIT), Electronic Materials Research Complex, 130 Samsung-ro, Gyeonggi-do, 16678, Republic of Korea; 3 CAE Group, Samsung Advanced Institute of Technology (SAIT), Electronic Materials Research Complex, 130 Samsung-ro, Gyeonggi-do, 16678, Republic of Korea

## Abstract

The solvent evaporation method on the structural changes and surface chemistry of the cathode and the effect of electrochemical performance of Li_1.0_Ni_0.8_Co_0.15_Mn_0.05_O_2_ (NCM) has been investigated. After dissolving of Li residuals using minimum content of solvent in order to minimize the damage of pristine material and the evaporation time, the solvent was evaporated without filtering and remaining powder was re-heated at 700 °C in oxygen environment. Two kinds of solvent, de-ionized water and diluted nitric acid, were used as a solvent. The almost 40% of Li residuals were removed using solvent evaporation method. The NCM sample after solvent evaporation process exhibited an increase in the initial capacity (214.3 mAh/g) compared to the pristine sample (207.4 mAh/g) at 0.1C because of enhancement of electric conductivity caused by decline of Li residuals. The capacity retention of NCM sample after solvent evaporation process (96.0% at the 50th cycle) was also improved compared to that of the pristine NCM sample (90.6% at the 50th cycle). The uniform Li residual layer after solvent treated and heat treatment acted like a coating layer, leading to enhance the cycle performance. The NCM sample using diluted nitric acid showed better performance than that using de-ionized water.

The demand for Li-ion batteries (LIBs) has increased rapidly owing to the high energy density of this battery system. As a result, LIBs have emerged as attractive energy sources for a variety of applications ranging from mobile products to devices with high energy capacity requirements such as electric vehicles (EVs) and hybrid electric vehicles (HEVs)^1^,^2^,^3^,^4^. For range-extended EVs, i.e., ones that can travel over 400 km on one full charge, power sources with high energy densities and excellent cycling lives are essential. However, conventional LIBs, which are composed of graphite as the anode and LiCoO_2_ as the cathode, cannot adequately meet the energy density requirements of range-extended EVs.

A significant amount of research has been conducted to improve the specific capacities of various cathodes^1^,^2^,^5^,^6^,^7^. For achieving capacity improvements, various types of transition metal (TM) oxides of the form LiMO_2_ (where M is a combination of TMs such as Ni, Co, and Mn) as well as spinel-structured compounds (e.g., LiMn_2_O_4_ and LiNi_0.5_Mn_1.5_O_4_) with high operating voltages have been considered as potential cathode materials. Specifically, Ni-rich materials with Ni content greater than 80% are considered to be the most favorable cathode materials for EV applications owing to their high capacity (~200 mAh/g) and low manufacturing costs^8^,^9^,^10^,^11^. However, major drawbacks such as structural degradation, gas evolution, cation mixing, and the presence of a large amount of Li_2_CO_3_ or LiOH (Li residuals) on the surface, have to be overcome before these materials can be successful commercialized^12^,^13^. Among these issues, the removal of Li residuals from Ni-rich materials is one of the key problems that need to be solved because the presence of Li residuals poses the potential risk of gas evolution on the surface and easy gelation of the cathode slurry by the polymerization of N-methyl-2-pyrrolidone (NMP). Additionally, LiOH reacts with LiPF_6_ in the electrolyte to form HF^10^,^14^.

An effective method to slow down the rates of these processes is to create an artificial physical barrier. The protective layer must be thick enough to avoid contact with NMP or the electrolyte. In addition, the thickness of the protective layer must be maintained within a few nanometers in order to enable Li^+^ diffusion and to achieve reasonable electronic conductivity with minimum capacity loss. In order to form the physical barrier and suppress the reaction between the cathode and electrolyte, inert materials such as Al_2_O_3_ and ZrO_2_ are generally coated on the surface of the cathode^15^,^16^. However, these materials lead to loss of capacity and lower the rate performance of the electrode because of the poor electronic conductivity of the coated material. Moreover, surface coatings are incapable of removing the Li residuals and the Li residuals get covered by the coating material. Another method for inhibiting the undesirable reactions involves re-heating of the cathode. Re-heating in an oxygen environment can remove a portion of the Li residuals. However, simply re-heating alone leads to a very small oxygen-rich stoichiometry in the structure and results in capacity deterioration^10^,^14^,^17^. The other method involves washing and filtering of the cathode powder. Washing in deionized (DI) water is found to be a very effective method for removing Li residuals. After filtering, the powder is re-heated in order to minimize the capacity drop^14^,^17^,^18^. Although the Li residuals are effectively removed using the washing and filtering method, a significant decline in the electrochemical performance is seen. Many research groups have also investigated methods for minimizing the performance drop by effectively removing Li residuals^7^,^9^,^11^,^14^,^17^,^18^,^19^.

The purpose of this study is to examine the role of the solvent evaporation method in effectively removing Li residuals and enhancing the electrochemical performance of LIBs containing Li_1.0_Ni_0.8_Co_0.15_Mn_0.05_O_2_ (NCM) as the cathode. After dissolving the Li residuals using the minimum possible amount of solvent in order to minimize damage to the pristine material as well as evaporation time, the solvent was evaporated at 120 °C without filtering. The remaining powder was re-heated at 700 °C in an oxygen environment. The influence of the solvent evaporation method on the structure and surface chemistry of the cathode was verified using X-ray diffractometry (XRD), high-resolution transmission electron microscopy (HRTEM), time-of-flight secondary ion mass spectroscopy (ToF-SIMS), and Auger electron spectroscopy (AES).

## Experimental

### Sample preparation

NCM was synthesized by means of the co-precipitation procedure. Suitable amounts of precursors of Ni, Mn, and Co (Ni/Co/Mn = 80:15:5) were dissolved in DI water and stirred to obtain a homogeneous solution. Next, a chelating agent along with a stoichiometric amount of NaOH solution was added with adequate stirring to achieve co-precipitation. The co-precipitated (NiMnCo)(OH)_2_ was co-ground with a stoichiometric amount of LiOH·H_2_O. Subsequently, NCM pristine material was obtained after the ground material was calcined at 750 °C for 24 h in O_2_. For the solvent evaporation process, 30 g of NCM powder was dissolved in 13 g of solvent. Two kinds of solvent were used for checking the effect of pH of solvent. The first one was DI water, and the other was a mixture of DI water and dilute nitric acid (DI water/nitric acid = 9:1). The minimum amount of solvent required for solvent evaporation process was determined based on Brunauer–Emmett–Teller (BET) analysis. For acid evaporated NCM and water-evaporated NCM, the same amount of solvent was used. The solution of NCM powder and solvent was stirred for 5 min and dried at 120 °C until the solvent completely evaporated. The acid evaporated NCM and water-evaporated NCM were prepared after the solvent dried NCM powders were heated at 700 °C for 5 h under O_2_ flow. Re-heated NCM was obtained after NCM pristine powder was heated again at 700 °C for 5 h under O_2_ flow without any treatment in order to check the effect of second heat treatment.

The Li, Mn, Co, and Ni concentrations in NCM were determined using inductively coupled plasma-atomic emission spectrometry (ICP-AES) on a Shimadzu ICPS-8100 sequential spectrometer. To avoid external contamination and oxidation, all the samples were transferred under an inert gas environment from the glove box to the analysis equipment using a specialized transfer vessel. The morphological changes of the NCM samples under various surface modification conditions were determined using SEM (Hitachi S-4700N). TEM images were obtained using an FEI Titan Cubed 60–300 microscope equipped with Cs correctors and a monochromator at an acceleration voltage of 300 V. The amounts of Li residuals were measured by titration. Since Li_2_CO_3_ and LiOH are also dissolved in water, most of the Li sources are believed to originate from LiOH and Li_2_CO_3_. The combined attributes of high spatial resolution (<10 nm) and near-surface (<50 Å) chemical information makes AES a powerful tool for comprehensive surface analysis. AES measurements were performed in an ultrahigh vacuum (UHV, 1 × 10^−8^ Pa) chamber at room temperature using a Scanning Auger Microscope (SAM, ULVACPHI PHI 710) with a cylindrical mirror analyzer and field emission electron gun at an acceleration voltage of 10 keV. The powders were also examined using a ToF-SIMS surface analyzer (TRIFT V, Ulvac PHI, Japan) operated at a pressure of 10^−9^ Torr, equipped with liquid Bi^+^ ion source and pulse electron flooding. During the analysis, the targets were bombarded with the 30 keV Bi^+^ beams with a pulsed primary ion current of −1 pA.

### Electrochemical measurements

Composite positive electrodes comprising of 92 wt.% active materials, 4 wt.% Denka black, and 4 wt.% polyvinylidene difluoride (PVDF) were pasted on an aluminum foil, which was used as the current collector. The electrodes were dried at 120 °C in vacuum and pressed. Metallic lithium was used as the counter electrode. The electrolyte solution consisted of 1.3 mol L^−1^ LiPF_6_ dissolved in an electrolyte based on ethylene carbonate (EC), dimethyl carbonate (DMC), and ethylmethyl carbonate (EMC). CR2032-type coin cells were assembled in a dry room. The loading level was 10 mg/cm^2^. For the galvanostatic experiments, the cells were discharged/charged galvanostatically at a constant current. Further, the electrochemical activities of the cathodes were characterized by electrochemical impedance spectroscopy (EIS) using a Solartron 1260 frequency-response analyzer. The impedance measurements were conducted over an applied frequency range of 10 mHz to 1 MHz at 25 °C. The EIS measurements were performed on the assembled cells at the initial state as well as discharged state after 50 cycles.

### Calculations

The equilibrium constant K for the reactions shown in Eqs 1 and 2 were calculated using the standard Gibbs free energy values referenced from the JANAF-NIMS thermochemistry data table^20^. The Gibbs free energy change of reaction, *ΔG*, can be expressed by Eqs 3 and 4 (derived in relation to Eqs 1 and 2), where *R* is the universal gas constant, T is the temperature, and *p*_*A*_ is the partial pressure of gas A. In reactions (1) and (2), only one of the constituents is in the gas phase, whereas the others are condensed phases. Therefore, ln(*p*_*A*_) for the condensed phases can be neglected and the calculated *K* is equal to the partial pressure of the gas in each reaction.


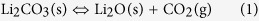



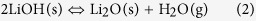



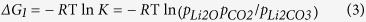



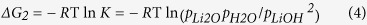


## Results and Discussion

**Figure 1:**
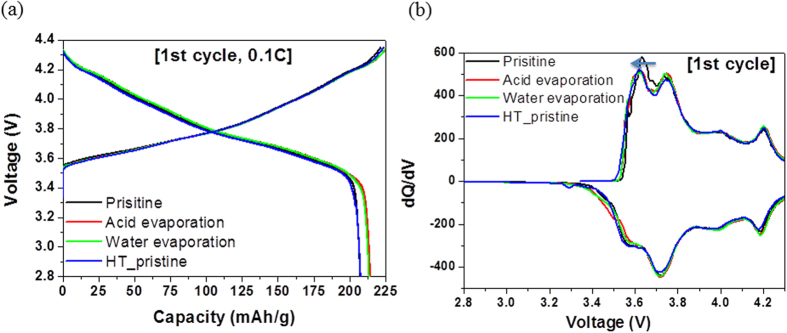
Comparison of (**a**) charge-discharge characteristics at the first cycle and (**b**) dQ/dV plot of pristine NCM and NCMs subjected to solvent evaporation.

Figure 1(a) shows the potential versus capacity profiles of pristine and solvent-treated NCM samples measured during the 1st cycle at a rate of 0.1C (0.175 mA/cm^2^). At a cutoff voltage of 2.5 V, the cells containing pristine, acid-treated, water-treated, and re-heated NCMs deliver reversible capacities of ~207.42, ~214.34, ~213.47, and ~207.6 mAh/g, respectively. The solvent-treated samples exhibit a higher capacity than the pristine samples. Moreover, the initial coulombic efficiency of the solvent-treated sample is higher than that of the pristine sample, which might be due to the enhanced reaction kinetics resulting from the improved electronic conductivity in the former case. The improved electronic conductivity is confirmed by the decrease in the charging voltage (red arrow) in the dQ/dV graph, as shown in Fig. 1(b). Meanwhile, the increase in capacity is not significant in the case of the re-heated sample.

**Figure 2:**
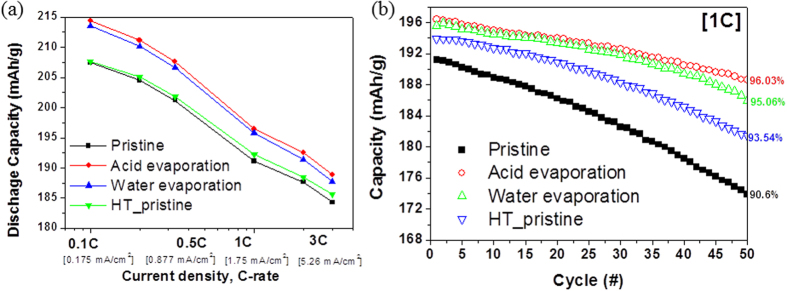
Comparison of (**a**) rate capabilities and (**b**) cycling performances of pristine NCM and NCMs subjected to solvent evaporation.

Next, the rate capabilities of all the samples are evaluated by cycling at various rates from 0.1C (0.175 mA/cm^2^) to 3 C (5.26 mA/cm^2^), and the results are presented in Fig. 2(a). As the applied current is increased, the capacities of all the samples decrease. Despite the high applied current density (more than 0.2C), the capacity values for the solvent-treated samples remain high owing to the improved electronic conductivity. The re-heated sample exhibits a slight improvement in performance at a high applied current density owing to the decrease in the amount of Li residuals. Figure 2(b) compares the cycling performance of the cells, at 25 °C, at a constant rate of 1C (1.75 mA/cm^2^). The capacity retention values are 90.6%, 96.03%, 95.06%, and 93.54% at the 50^th^ cycle for the pristine, acid-treated, water-treated, and re-heated cathodes, respectively. The capacity retention was also enhanced after reheating without any solvent treatment. All the solvent-treated cathodes exhibit higher capacity retention during cycling compared to the pristine cathode. More importantly, the solvent-treated samples exhibit better retention rate compared to the re-heated sample. Generally, besides Li residuals, TMs are also removed when acid is used as the solvent. However, in the present study, the acid-treated sample exhibits better electrochemical performance compared to pristine NCM.

**Figure 3:**
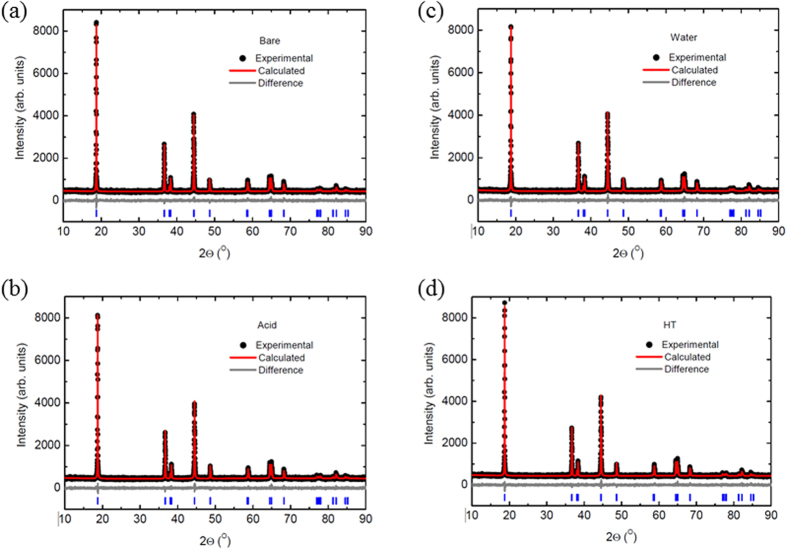
Rietveld refinement results calculated from the XRD data of various samples (**a**) pristine NCM sample, (**b**) acid-treated sample, (**c**) water-treated sample, and (**d**) re-heated sample.

To evaluate the composition changes during the solvent evaporation process, inductively coupled plasma mass spectroscopy (ICP-MS) measurements are conducted. The Li, Ni, Mn, and Co content ratios are found to be nearly identical within the tolerance ranges of the equipment for all the samples because filtering has not applied in the sample preparation and any elements have not removed through the solvent evaporation process, as shown in Supplementary Information. Figure 3 shows the Rietveld refinement results calculated from the XRD data for the pristine and solvent evaporated NCM samples. All the diffraction peaks are assigned based on the hexagonal α-NaFeO_2_ structure (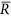 3*m*). Phases containing impurities are not considered in this assignment. It should be noted that re-heating NCM at 700 °C without any added solvent does not alter the crystal structure, as shown in Fig. 3(d). As shown in Table 1, further analysis of the lattice parameters reveals that neither evaporation nor re-heating affects the lattice parameters of the pristine sample. It is well-known that among Ni, Co, and Mn, Ni ions can easily occupy the lattice sites of Li ions because the ionic radius of Ni^2+^ (0.69 Å) is close to that of Li^+^ (0.76 Å). This phenomenon is known as “cation mixing”^3^,^4^,^5^,^10^. Therefore, when the Ni content increases, the extent of cation mixing is likely to increase^5^,^10^. On the other hand, Li ions, which do not occupy their original site, adhere to the surface of the cathode in the form of Li_2_CO_3_/LiOH (referred as Li residuals)^10^,^14^,^21^. Moreover, in the case of Ni-rich (Ni content >80%) NCM, excess Li is required for achieving satisfactory performance during the synthesis of Ni layered cathode material. This excess Li can also participate in the formation of Li residuals on the surface of NCM. As shown in Table 1, the amount of Ni^2+^ in the Li layer slightly decreases when the NCMs are subjected to solvent evaporation. The Ni^2+^ contents in the pristine sample, sample subjected to acid evaporation, and sample subjected to water evaporation are 3.09%, 2.96%, and 2.82%, respectively.

**Figure 4:**
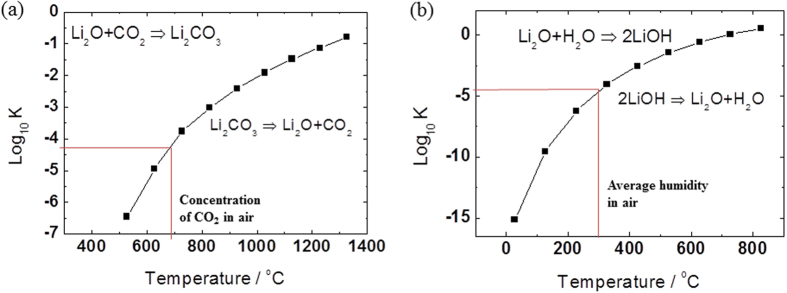
Phase diagram of Li_2_CO_3_ and LiOH as a function of temperature.

The amount of Li residuals is closely related to the cation mixing behavior because the unoccupied Li ions in Li layer can be transformed to Li residuals. The amount of Li residuals in the pristine and coated samples is listed in Table 2. It is interesting to note that the total amount of Li residuals in the re-heated NCMs subjected to solvent evaporation decreases. However, the decrease in the amount of Li residuals in the re-heated sample is much smaller than that in the other samples (water/acid evaporated samples), implying that re-heating NCM materials is not an effective method for eliminating Li residuals. Our previous results suggest that re-heating NCMs in air leads to an increase in the amount of Li residuals owing to the reaction between Li and CO_2_/H_2_O present in air. However, since the re-heating treatment is conducted under an O_2_ environment in this study, the amount of Li residuals does not increase owing to the insufficient amounts of CO_2_ and H_2_O. On the other hand, some of the Li generated from decomposed Li residuals can be relocated in the Li layer of NCM or may react with the decomposed TMs via solvent mixing. The relocation of Li in the Li layer by heat treatment is confirmed by the decrease in the proportion of Ni^2+^ in the Li layer for the solvent-treated samples, as shown in Table 1. To further support this argument, the decomposition reactions of Li_2_CO_3_ and LiOH are obtained in Fig. 4. The reactions indicate that Li_2_CO_3_ may decompose to form Li_2_O and CO_2_ depending on the temperature and partial pressure of CO_2_ in the reaction environment, whereas LiOH may decompose to form Li_2_O and H_2_O. In the present study, Li_2_CO_3_ and LiOH are decomposed at a re-heating temperature of 700 °C under ambient conditions and average atmospheric humidity. Since Li_2_CO_3_ and LiOH begin to thermally decompose to Li_2_O beyond 500 °C, it is assumed that the reacting Li material is mainly composed of Li_2_O. It is important to note that the NiO phase of the rock salt structure is considerably developed on the surface of Ni-rich NCM, as confirmed by Shim *et al*.^22^. Hence, it is highly probable that NiO reacts with the residual Li_2_O to produce lithium nickel oxide (LiNiO_2_), as demonstrated by Wang *et al*. using DFT calculations^23^.


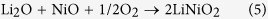


**Figure 5:**
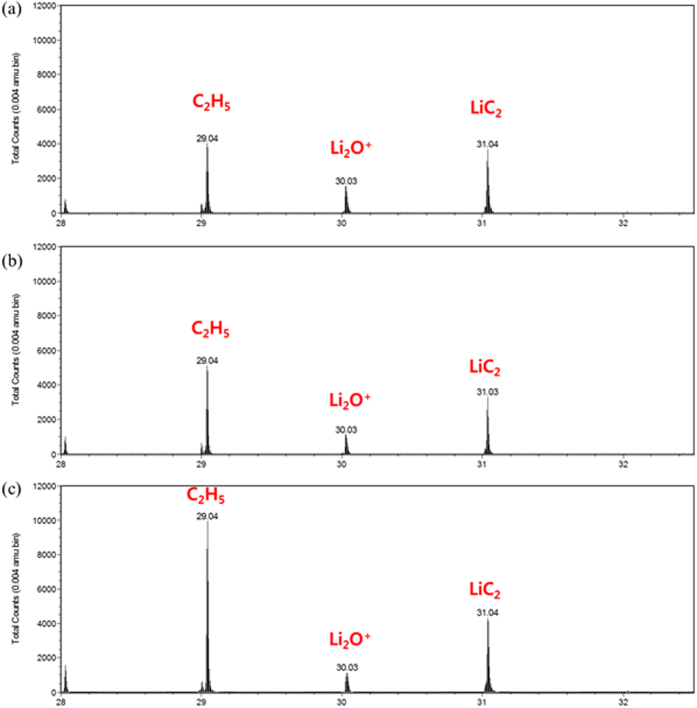
ToF-SIMS spectra showing peaks for Li_2_O^+^ and LiC_2_^+^ in the (**a**) pristine sample, (**b**) acid-evaporated NCM, and (**c**) water-evaporated NCM.

The Li remaining after relocation and reaction with the TMs can produce Li residuals again owing to its reaction with CO_2_ and H_2_O in the air. To further confirm the change in the amount of Li residuals, ToF-SIMS analysis is conducted in Fig. 5. Besides Li residuals, a portion of Li in the layered oxide is measured using the titration method. However, the ToF-SIMS data only provide qualitative information on the accumulation of Li compounds on the surface. The ToF-SIMS peaks at m/z values of ~30.03 and 31.04 shown in Fig. 5 are assigned to Li_2_O^+^ and LiC_2_, respectively. After solvent evaporation, the intensities of the two peaks (measured for two samples) decrease as compared to that for the pristine sample (Table 3)^10^. Titration experiments reveal that among the Li residuals, only Li_2_CO_3_ shows a decrease in concentration. However, in addition to Li_2_CO_3_, LiOH is removed from the surface, as shown by the ToF-SIMS data. It is also found that acid is a more effective solvent for the removal of Li_2_CO_3_ than water.

**Figure 6:**
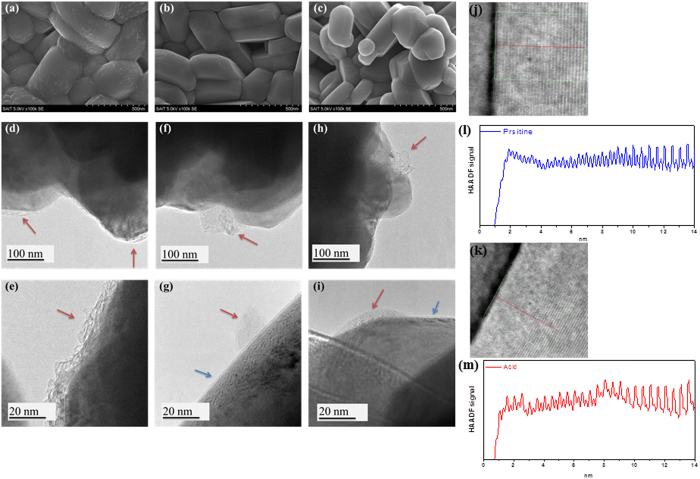
SEM images of (**a**) pristine NCM, (**b**) acid-evaporated NCM, and (**c**) water-evaporated NCM. HRTEM images of (**d**,**e**) pristine NCM, (**f**,**g**) acid-evaporated NCM, and (**h**,**i**) water-evaporated NCM. HADDF-STEM images of (**j**) pristine NCM and (**k**) acid evaporated NCM. HADDF-STEM signal profiles of (**l**) pristine NCM and (**m**) acid-evaporated NCM from the areas outlined in the HAADF-STEM images.

Changes in the morphology of the Li residuals during the course of the solvent evaporation process are analyzed using SEM and TEM imaging; the images are shown in Fig. 6. Notably, the surface morphologies of the pristine samples and samples treated by solvent evaporation are quite different. The surface of the pristine sample is observed to be slightly rough and mostly covered by island-like particles, which could be Li residuals. On the other hand, the surface of the samples treated by the solvent evaporation method is found to be clean, indicating that a large portion of the Li residuals are washed away or transformed to other compounds such as LiNiO_2_ during the treatment.

Figure 6(d–i) shows the TEM images. As observed in the Fig. 6(e), the surface of the pristine sample is covered with a 10 nm thick layer of Li residuals. However, island-type Li residuals (red arrow), and a very thin (2 nm) and uniform layer of Li residuals (blue arrow) are detected in the images of the samples treated by solvent evaporation. The very thin and uniform layer of Li residuals might result from the reaction between Li_2_O and a small amount of CO_2_/H_2_O, which may exist in the O_2_ gas at reduced temperatures.

High-angle annular dark-field scanning transmission electron microscopy (HAADF-STEM) imaging reveals that the samples consist of a Li layer and TM layer along the c axis in Fig. 6(j–m) 8. Analysis of two samples reveals a two-dimensional ordered structure with perfect Li and TM ordering and no defects between the Li and TM layer within the sample. However, the surface observed in the HAADF-STEM images provides direct evidence for the structural changes and the mixing of Li and TM. The observed structure might be due to cation mixing, or may be the rock salt structure^8^. In the case of the pristine sample, it is difficult to distinguish between the Li and TM layers up to 10 nm because of the mixing of these two layers, as shown in Fig. 6(j,k). On the other hand, it is possible to identify the Li and TM layers in the treated sample, although the intensity of HADF signal is changed in this case. Therefore, it is highly probable that LiNiO_2_ is formed on the surface during the solvent evaporation process.

**Figure 7:**
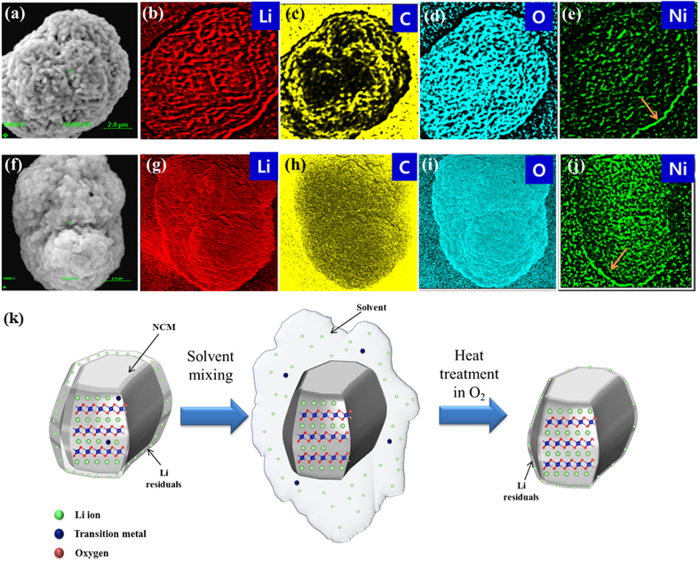
Auger maps showing the qualitative distribution of Li, C, O, and Ni, (**a**–**e**) pristine and (**f**–**j**) acid-evaporated NCM. (**k**) Schematic description of the solvent evaporation process.

The combination of high spatial resolution and near-surface chemical information makes AES a powerful tool for comprehensive surface analysis. Figure 7 shows the elemental mapping images of the pristine and solvent-treated NCM samples obtained by AES. The NCM samples are treated with acid during the solvent treatment procedure. A significant amount of carbon is found to be agglomerated on the surface and covers the surface in the case of pristine NCM. This carbon might originate from Li_2_CO_3_. On the other hand, the carbon on the surface of the solvent-treated NCM sample is well-distributed, and its total content is less than that on the pristine surface. This result also indicates that the Li residuals are removed and a thin and uniform Li layer is formed during the solvent treatment process. Moreover, the Ni content at the edge of the two samples (orange arrow in Fig. 7 is higher than that in the bulk. The higher distribution of Ni at the edge might arise from NiO, which is formed during the sintering process^22^. NiO can react with the residual Li_2_O to produce LiNiO_2_, as previously shown in Eq. (5). To better understand the mechanism of removal of Li residuals using the solvent evaporation method, a schematic illustration is presented in Fig. 7(k).

**Figure 8:**
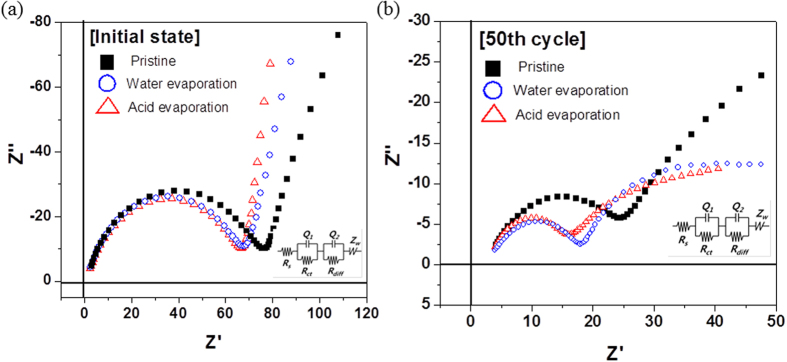
EIS spectra of the pristine NCM and NCMs subjected to solvent evaporation measured at 25 °C (**a**) initial state and (**b**) after 50 cycles.

As mentioned in the previous section, the presence of a thin and uniform layer of Li residuals is confirmed on the surface of the sample after the solvent-treatment process. The Li residuals consist of inert materials such as Al_2_O_3_ or ZrO_2_, which act as coatings to prevent any interactions between the cathode and the electrolyte. Hence, it is probable that the uniform Li residual layer formed by solvent and heat treatments acts as a coating layer, leading to enhanced cycle performance. To confirm the thin coating layer effect, EIS test is conducted at 25 °C. The EIS results of initial state and discharged state after 50 cycles are shown in Fig. 8. The solvent-treated material showed a lower impedance at the initial state because of reduced resistance caused by the removal of Li residuals. After the 50^th^ charge/discharge cycle, the impedances of solvent-treated NCMs are much smaller than that of pristine, which is related to the prevention of reaction between cathode and electrolyte^24^,^25^. That is similar to effect of inert coating layer^15^,^26^. The formation of uniform Li residual layer formed by solvent and heat treatments is confirmed again.

## Conclusion

In this study, the effect of solvent evaporation treatment on the structural changes and surface chemistry of the Li_1.0_Ni_0.8_Co_0.15_Mn_0.05_O_2_ (NCM) cathode was investigated using XRD, HRTEM, ToF-SIMS, and AES characterization techniques. The electrochemical performance of the treated cathode was systematically characterized by electrochemical measurements using 2032 coin cells. Nearly 40% of the Li residuals was effectively removed using the solvent evaporation method. The possibility of LiNiO_2_ formation on the surface was verified using AES and HAADF-STEM analysis. The NCM sample obtained after the solvent evaporation process exhibited an enhanced initial capacity (214.3 mAh/g) compared to the pristine sample (207.4 mAh/g) at a charge/discharge rate of 0.1C, owing to enhancement in the electric conductivity caused by a decrease in the amount of Li residuals and formation of LiNiO_2_. The capacity retention of the NCM sample after the solvent evaporation process (96.0% at the 50^th^ cycle) was also improved compared to that of the pristine NCM sample (90.6% at the 50^th^ cycle). The uniform Li residual layer observed after solvent and heat treatments acted like a coating layer, leading to enhanced cycle performance. The sample treated with dilute nitric acid exhibited better electrochemical performance among the samples considered in this study, owing to minimized TM and impurity removal from the surface as a result of the short mixing time (5 min).

## Additional Information

**How to cite this article**: Park, K. *et al*. Re-construction layer effect of LiNi_0.8_Co_0.15_Mn_0.05_O_2_ with solvent evaporation process. *Sci. Rep.* **7**, 44557; doi: 10.1038/srep44557 (2017).

**Publisher's note:** Springer Nature remains neutral with regard to jurisdictional claims in published maps and institutional affiliations.

## Supplementary Material

Supplementary Information

## Figures and Tables

**Table 1:** Rietveld analysis results for the pristine NCM and NCM samples subjected to solvent evaporation.

Samples	Ni^2+^ in the Li layer (%)	*a* (Å)	*b* (Å)	*R*_*wp*_/*%*
Pristine	3.09	2.8675	14.1854	4.920
Acid-treated	2.96	2.8691	14.1897	4.740
Water-treated	2.82	2.8687	14.1884	4.875
Re-heated	3.07	2.8675	14.1857	4.865

**Table 2:** Concentration of Li residuals in the pristine NCM and NCM samples subjected to solvent evaporation.

Samples	Li_2_CO_3_ (ppm)	LiOH (ppm)	Total (ppm)
Pristine	9561	6555	3693
Acid-treated	2890	5800	2222
Water-treated	2190	6070	2168
Re-heated	8840	5920	3374

**Table 3:** ToF-SIMS intensity ratios normalized by the total contents for pristine NCM and NCM samples subjected to solvent evaporation.

Samples	Li_2_O	LiC_2_
Pristine	0.41	0.95
Acid-treated	0.28	0.53
Water-treated	0.27	0.74
