# Supplementary material for: Re-construction layer effect of LiNi0.8Co0.15Mn0.05O2 with solvent evaporation process
Source: Sci Rep. 2017 Mar 20;7:44557. doi: 10.1038/srep44557 (PMC5357953; doi:10.1038/srep44557)
Supplement: Supplementary Information [file srep44557-s1.pdf]

Supplementary Information

Re-construction layer effect of $LiNi_{0.8}Co_{0.15}Mn_{0.05}O_2$ with solvent evaporation process

Kwangjin Park[a*], Jun-Ho Park[a], Suk-Gi Hong[a], Byungjin Choi[a], Sung Heo[b], Seung-Woo Seo[c], Kyoungmin Min[c*], Jin-Hwan Park[a]

[a]Energy Lab, Samsung Advanced Institute of Technology (SAIT), Electronic Materials Research Complex, 130 Samsung-ro, Gyeonggi-do, [16678], Republic of Korea

[b]Platform Technology Lab, Samsung Advanced Institute of Technology (SAIT), Electronic Materials Research Complex, 130 Samsung-ro, Gyeonggi-do, [16678], Republic of Korea

[c]CAE Group, Samsung Advanced Institute of Technology (SAIT), Electronic Materials Research Complex, 130 Samsung-ro, Gyeonggi-do, [16678], Republic of Korea

SI. ICP results of pristine samples and NCMs subjected to solvent evaporation

Sample	Mole ratio, %			
	Li	Mn	Co	Ni
Pristine	1.06	0.05	0.148	0.802
Acid-treated	1.04	0.05	0.149	0.801
Water-treated	1.05	0.05	0.149	0.801
